# Supplementary material for: HIVprotI: an integrated web based platform for prediction and design of HIV proteins inhibitors
Source: J Cheminform. 2018 Mar 9;10:12. doi: 10.1186/s13321-018-0266-y (PMC5845081; doi:10.1186/s13321-018-0266-y)
Supplement: Supplementary file 2 — Additional file 2. Source code of HIVProtI web server. [file 13321_2018_266_MOESM2_ESM.zip › HIVprotI_Source-code/hivprotI/jsmol/sync2.htm]

|  |  |  |
| --- | --- | --- |
| Synchronize  File-derived structure      Jmol-derived structure | This page demonstrates how to synchronize applets in Jmol. The two applets are jmolAppletA and jmolAppletB. Once the checkbox is checked, going to either one controls the other. The command used here is simply:  ``` function sync() {  var syncing = document.getElementById("drive").checked  var s = (syncing ? 'sync * on; sync * "set syncMouse true"': 'sync * off')  jmolScript(s, "A"); } ```  Alternatively, we could have used:    `jmolScript("sync . ON;sync > SLAVE", "A")`    which would cause only the applet on the top to do the controlling, but allowed the bottom applet to be moved independently of that control. Also, if you open a console on one, you can give commands to the other using something like:  ``` sync > "background black" ```  The ">" means "all other synchronized applets". Other options include "\*" (all synchronized applets) and "." (only this applet, if it is synchronized). In addition, you can specify the applet by its extension, "A" or "B", or by its full name, "jmolAppletA" or "jmolAppletB" in this case.    Starting with Jmol 11.6, you can also use the **script** command with the APPLET option to send scripts to one or more applets. This operation is independent of the state of sync being ON or OFF.  ``` script APPLET * "background yellow" ```     Also new in Jmol 11.6, you can use the Jmol math script() function to get information from another applet. Simply indicate the target applet as a second parameter to the script command. For example, from Applet A's console, type:  ``` print script("show orientation","B") ``` |  |
| ``` Jmol version=11.3.9  # new feature: synchronization of applets using Jmol scripts: # # sync .\|>\|*\|appletId[syncId] ON\|OFF\|SLAVE\|command # # The sync command allows two or more applets to be synchronized in # terms of orientation. Move one with the mouse, and the other moves as well. # In addition, the sync command allows ANY command to be sent to one or more # other applets directly, without the intervention of JavaScript. # # Applets are identified by appletId (jmolApplet0, for instance) # along with an optional bracketed sync group identifier -- generally a random  # number that identifies the page containing the controlling applet. If the  # syncId is not given, then the ID for the page containing the controlling applet # is used. This feature is important for cross-frame synchronization only.  # # # .          this applet only # >          all applets except this one # *          all applets # appletId   id of a specific applet # [syncId]   (optional) a unique string of digits -- brackets included # # ON         sync as driver (default) # OFF        turn sync off # SLAVE      turn sync on, but not as driver # command    command to send # # for example: # # sync *   # synchronize all applets as drivers # sync jmolApplet1  #syncs this applet with jmolApplet1 both as drivers # sync > "set echo top left;echo OK"  # sends OK to top left of all OTHER applets # sync jmolApplet2[254678942] OFF  # turns sync off for an applet ON A DIFFERENT PAGE #                                  # or in a different FRAME # sync . OFF # turns sync off for this applet # # new Jmol.js feature: jmolGetSyncId(); jmolSetSyncId(id); # allows control over the sync ID via javascript. jmolSetSyncId(id) # should be called prior to jmolApplet() and should incorporate some sort of # random digits and no space characters. (A number is good.) # This should only be necessary for multi-frame pages.  # ----------------------------------------------------------------------------- ``` | | |
